# Diabetic Nephropathy: Novel Molecular Mechanisms and Therapeutic Targets

**DOI:** 10.3389/fphar.2020.586892

**Published:** 2020-12-21

**Authors:** Carlamaria Zoja, Christodoulos Xinaris, Daniela Macconi

**Affiliations:** ^1^ Istituto di Ricerche Farmacologiche Mario Negri IRCCS, Centro Anna Maria Astori, Science and Technology Park Kilometro Rosso, Bergamo, Italy; ^2^ University of Nicosia Medical School, Nicosia, Cyprus

**Keywords:** diabetic nephropathy, renin-angiotensin system, angiotensin 1–7, sirtuins, notch signaling, thyroid hormone signaling, sodium-glucose cotransporter 2, hypoxia inducible factor

## Abstract

Diabetic nephropathy (DN) is one of the major microvascular complications of diabetes mellitus and the leading cause of end-stage kidney disease. The standard treatments for diabetic patients are glucose and blood pressure control, lipid lowering, and renin-angiotensin system blockade; however, these therapeutic approaches can provide only partial renoprotection if started late in the course of the disease. One major limitation in developing efficient therapies for DN is the complex pathobiology of the diabetic kidney, which undergoes a set of profound structural, metabolic and functional changes. Despite these difficulties, experimental models of diabetes have revealed promising therapeutic targets by identifying pathways that modulate key functions of podocytes and glomerular endothelial cells. In this review we will describe recent advances in the field, analyze key molecular pathways that contribute to the pathogenesis of the disease, and discuss how they could be modulated to prevent or reverse DN.

## Introduction

Diabetes is a global epidemic that is creating an unsustainable strain on healthcare systems due to its rising incidence worldwide and the costs associated with its chronic complications *(* http://www.idf.org/diabetesatlas *)*. About one-third of diabetic patients develop diabetic nephropathy (DN), which in patients with micro- and then macro-albuminuria tends to progress to end-stage renal disease (ESRD) (Remuzzi et al., 2002). In type 2 diabetes, albuminuria is now recognized not simply as a marker of renal dysfunction but also as a risk factor for cardiovascular disease, which is three times as high as that for diabetic patients with no evidence of renal disease (Fox et al., 2004; Zhou et al., 2009). Renin-angiotensin system (RAS) inhibitors reduce albuminuria and the cardiovascular complications of diabetes but may provide incomplete renoprotection if started late in the course of the disease (Perico et al., 1994; Ruggenenti et al., 2010). Developing efficient therapies for DN is extremely challenging because of the complex pathobiology of the diabetic organ, which undergoes a set of profound structural, metabolic and functional changes.

*Glomerular visceral epithelial cells (podocytes).* Podocytes are the main determinant of the maintenance of the perm-selective properties of the glomerular filtration barrier (Nagata, 2016; Conti et al., 2017), and podocyte dysfunction has been considered a major factor in the development of diabetic glomerular disease (Pagtalunan et al., 1997; Wolf et al., 2005). Podocytes are highly specialized cells located on the visceral side of the Bowman’s capsule and exhibit podocyte foot processes, which are connected by a specialized intracellular junction, the slit diaphragm, which in turn forms a size-selective barrier for the passage of large molecules. Specific diaphragm proteins, such as nephrin, form the filtration slits (Conti et al., 2017). At the slit diaphragm, podocin and other proteins provide structural and functional support to the filtration barrier and participate in signaling pathways by interacting with actin cytoskeleton components (Perico et al., 2016a; Schell and Huber, 2017). Reduced expression of podocyte proteins, which reflects podocyte dysfunction, and a reduced number of podocytes are characteristic features of DN, both in experimental models and humans (Pagtalunan et al., 1997; Aaltonen et al., 2001; Benigni et al., 2004; Lin and Susztak, 2016). A correlation between podocyte detachment/loss and the albumin excretion rate has been reported in DN patients (Pagtalunan et al., 1997; Meyer et al., 1999; Lemley et al., 2000). Recently, scanning electron microscopy (SEM) analysis of the podocyte cytoarchitecture in type 2 diabetic patients at different stages of kidney disease showed that in normoalbuminuric subjects, podocytes had intact cell bodies with normal interdigitating foot processes (Conti et al., 2018). In patients with micro-albuminuria, features of podocyte injury, consisting of podocyte hypertrophy with diffuse foot process effacement and occasional pseudocysts representing site of initial cell detachment from the GBM, were observed. In the late stages of proteinuric DN the structural integrity of the glomerular barrier was irreversibly compromised, with the occurrence of striking podocyte loss and extensively denuded glomerular basement membranes (Conti et al., 2018). These observations help explain why drugs may fail to affect renal disease progression in the latter circumstance while underlining the need for early therapeutic intervention to efficiently achieve renoprotection. Although podocytopathy has been considered the culprit in the development of diabetic glomerular disease, glomerular endothelial dysfunction also plays a key role in the pathogenesis and progression of DN (Toyoda et al., 2007; Broekhuizen et al., 2010; Kuwabara et al., 2010; Satchell, 2012; Weil et al., 2012).

*Glomerular endothelium.* The glomerular endothelium along with the glycocalyx–a negatively charged network of proteoglycans and glycoproteins that covers the luminal surface of fenestrated glomerular endothelial cells–has been recognized as crucial in restricting the passage of plasma proteins and preserving the glomerular filtration barrier (Fogo and Kon, 2010; Haraldsson and Nystrom, 2012; Salmon and Satchell, 2012). Loss of the endothelial glycocalyx is linked to increased vascular permeability in type 2 diabetic patients and to albuminuria in experimental DN (Broekhuizen et al., 2010; Kuwabara et al., 2010). Lower endothelial cell fenestrations are associated with macroalbuminuria and GFR decline (Weil et al., 2012) and glomerular capillary loss correlates with the degree of glomerulosclerosis (Hohenstein et al., 2006). Multiple pathways contribute to endothelial dysfunction in DN. Hyperglycemia and oxidative stress cause glycocalyx destruction through the induction of heparanase, a degrading enzyme of heparan sulfate, reduced synthesis of heparan sulfate, and uncoupling of the endothelial nitric oxide synthase (Fu et al., 2015; Jourde-Chiche et al., 2019). Studies have provided evidence that there is cross-talk between glomerular endothelial cells and podocytes that is important in regulating survival and function for both cells (Satchell, 2012; Fu et al., 2015; Lennon and Hosawi, 2016; Cassis et al., 2019b). Thus, glomerular endothelial dysfunction may cause injury in the neighboring podocytes and, *vice versa*, podocyte activation may foster endothelial damage through specific paracrine signals. Vascular endothelial growth factor (VEGF), angiopoietins, endothelin-1, transforming growth factor-β (TGF-β), to name a few, have all been implicated as major mediators of this vicious cycle (Fu et al., 2015; Garsen et al., 2016; Wu et al., 2017; Jourde-Chiche et al., 2019). Targeting the reciprocal interaction between endothelial cells and podocytes may be a therapeutic opportunity to limit DN progression.

Impairment of the glomerular filtration barrier, with the onset of overt proteinuria, accelerates the progression of diabetic kidney disease, and glomerular sclerosis and interstitial fibrosis is the final step toward ESRD. Several studies have elucidated the complexity of the fibrogenic process in the kidney, which involves the interplay among different cell types, the activation of several profibrotic pathways, including the most known TGF-β, and their epigenetic regulation (Srivastava et al., 2013; Lovisa et al., 2016; Macconi, et al., 2016; Zhao et al., 2020). Kidney fibrosis develops through intracellular mechanisms, that comprise glomerular and tubular epithelium distress, inflammation, dysregulated innate and adaptive immune response, tubular injury and atrophy, and microvasculature rarefaction (Liu, 2011; Tang and Yiu, 2020). In the last decade potential progenitors for myofibroblasts were identified which include proliferating resident interstitial fibroblasts, bone marrow-derived cells, perivascular mesenchymal stem cells, and epithelial and endothelial cells that acquire a myofibroblast phenotype in processes termed epithelial to mesenchymal transition (EMT) and endothelial-to-mesenchymal transition (EndMT) (Zeisberg et al., 2008; LeBleu et al., 2013; Srivastava et al., 2013; Falke et al., 2015; Kramann et al., 2015; Lovisa et al., 2016; Srivastava S. P. et al., 2019).

Experimental models of diabetes have revealed promising therapeutic targets by enabling the identification of pathways that modulate key functions of podocytes and glomerular endothelial cells. In this review, we describe recent advances in the field and discuss emerging therapeutic strategies. Specifically, we focus on the angiotensin converting enzyme 2 (ACE2)/Angiotensin-(1–7)/Mas receptor axis and the protective effects of cyclic Ang-(1–7) on both podocytes and glomerular endothelial cells in experimental type 2 DN. Morever, we discuss Sirtuins 1 and 3 as a therapeutic target for counteracting diabetes-induced oxidative stress and glomerular injury. The role of the developmental pathways Notch1 and thyroid hormone signaling in podocytes during diabetic disease is described as a mechanism that underlies podocyte de-differentiation and loss. Finally, the contributions of the sodium-glucose cotransporter 2 (SGLT2), hypoxia-inducible factor-1 (HIF-1) and dipeptidyl peptidase-4 (DPP-4) signaling pathways to the progression of DN are discussed.

## The Renin-Angiotensin System

### Angiotensin Converting Enzyme/Angiotensin-II/Ang II Type 1-Ang II Type 2 Receptors

**FIGURE 1:**
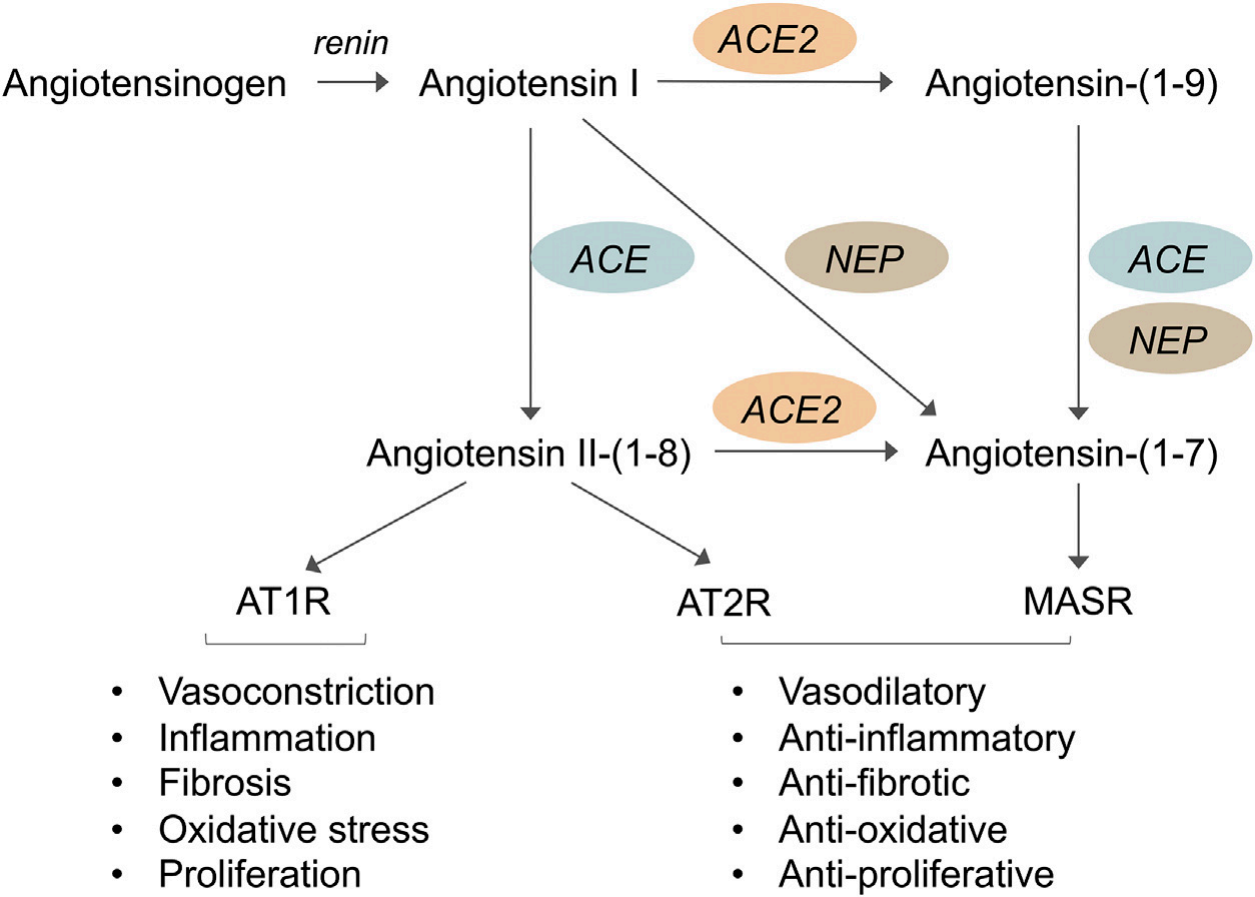
Schematic representation of the renin-angiotensin system (RAS) showing that the angiotensin converting enzyme 2 (ACE2)/Angiotensin-(1–7)/Mas receptor axis exerts opposite effects to those of ACE/Angiotensin II/AT1 receptor axis. Renin cleaves hepatic angiotensinogen into Angiotensin I which is then cleaved via ACE into Angiotensin II. The effects of Angiotensin II are exerted mainly through the activation of the Angiotensin II type 1 receptor (AT1R) and includes vasoconstriction, inflammation, fibrosis, oxidative stress and cell growth. Angiotensin II also binds to Angiotensin II type 2 receptor (AT2R) which usually opposes the actions of AT1R. Angiotensin-(1–7), which is a specific Mas receptor (MasR) agonist, can be formed directly from Angiotensin II via ACE2, or it can be generated through the ACE2-catalyzed hydrolysis of Angiotensin I to the inactive Angiotensin-(1–9) which is then converted to Angiotensin-(1–7) by ACE or neprilysin (NEP). However, Angiotensin-(1–7) is mainly formed through the action of ACE2 on Angiotensin II which has more affinity to ACE2 than Angiotensin I. When levels of Ang II are not sufficiently elevated, Ang-(1–7) can also be formed directly from Ang I via NEP. Interaction of Angiotensin-(1–7) with MasR triggers intracellular signaling pathways leading to beneficial actions such as vasodilation, anti-inflammatory, anti-fibrotic and anti-oxidative effects, and inhibition of cell proliferation.

The RAS plays a key role in a variety of physiological and pathological processes. The RAS is activated by the secretion of renin by the juxtaglomerular cells of the kidney. Renin hydrolyzes liver-derived angiotensinogen into angiotensin (Ang) I, a decapeptide, which is then cleaved by ACE into the octapeptide Ang II (Figure 1). This occurs not only in the circulation but also locally in several organs, including the kidney, blood vessels, and heart. The effects of Ang II are exerted mainly through the activation of the G protein-coupled receptor Ang II type 1 receptor (AT1R) and include vasoconstriction, fluid retention, inflammation, fibrosis, oxidative stress, and cell growth and migration, to name a few (Forrester et al., 2018).

Ang II also binds to Ang II type 2 receptor (AT2R), which usually has the opposite effects of AT1R, in terms of blood pressure regulation, vascular remodeling and cell growth (Horiuchi et al., 1999; Jones et al., 2008; Kaschina et al., 2017). The local actions of Ang II depend on the combined net effect of AT1R and AT2R, so that the levels of AT2R expression relative to AT1R in different pathological states, including diabetes, may be crucial for determining the end-organ response (Jones et al., 2008). However, the signaling mechanisms of AT2R are not completely understood (Forrester et al., 2018). While several studies have reported beneficial effects of AT2R activation on organ protection (Naito et al., 2010; Padia and Carey, 2013; Chow and Allen, 2016; Sumners et al., 2019), some others have shown that increased activation of AT2R could have detrimental effects (Cao, 2002; Tejera et al., 2004). Most of the RAS inhibitors currently used to delay the progression of kidney injury in diabetes target the ACE/Ang II/AT1 receptor axis.

### Angiotensin Converting Enzyme Substrate N-Acetyl-Seryl-Aspartyl-Lysyl-Proline has Antifibrotic Properties in Diabetic Nephropathy

ACE has two homologous N- and C-terminal active domains (Bernstein et al., 2011). While the ACE C-terminal catalytic domain is the main site of Ang I cleavage into Ang II *in vivo* (Fuchs et al., 2008), the N-domain specifically cleaves its natural substrate N-acetyl-seryl-aspartyl-lysyl-proline (Ac-SDKP) into inactive fragments (Fuchs et al., 2004). Ac-SDKP is released by the nephron from its precursor thymosin β4 through two-step proteolytic cleavage that involves meprin*α* and the serine protease prolyl oligopeptidase (POP), also known as prolyl endopeptidase (Prep) (Kumar et al., 2016), and the thymosin β4-Ac-SDKP axis is a peptidergic system that prevents kidney fibrosis under normal conditions (Romero et al., 2019) and can reduce established fibrosis during kidney injury (Zuo et al., 2013). The homeostatic role of Ac-SDKP in collagen balance is further supported by evidence that low endogenous levels of the peptide in the kidneys as a result of POP inhibition promote organ fibrosis, which is accelerated in the presence of profibrotic stimuli (Cavasin et al., 2007). Consistently, the epigenetic downregulation of POP/Prep by miR-324-3p contributed to kidney fibrosis in chronic kidney disease (CKD) by rendering renal tubular cells more prone to acquiring a mesenchymal phenotype in response to profibrotic stimuli (Macconi et al., 2012). The rise in plasma and urinary levels of Ac-SDKP caused by ACE inhibitors is part of their renoprotective effect (Macconi et al., 2012; Nagai et al., 2014; Srivastava et al., 2020a; Srivastava et al., 2020b). Differences in the renal protective ability of ACE inhibitors and AT1R blockers (ARB) have been described in experimental DN, which were attributable to the capacity of ACE inhibitor, but not ARB, to prevent ACE-induced Ac-SDKP degradation (Nagai et al., 2014; Srivastava et al., 2020a; Srivastava et al., 2020b). In a mouse model of type 1 DN, both ACE inhibitor and Ac-SDKP, but not ARB, ameliorated renal fibrosis by controlling the metabolic switch between glucose and fatty acid metabolism, thus suppressing glycolysis-related EMT (Srivastava et al., 2020b). Moreover, the ACE inhibitor alone or combined with Ac-SDKP inhibited the renal overexpression of the enzyme dipeptidyl peptidase-4 (DPP-4) and the activation of TGF-β signaling by restoring the expression of the anti-fibrotic microRNAs miR-29s and miR-let-7s, which targeted DDP-4 and the TGF-β receptor TβRI, respectively. This resulted in reduced EndMT and ECM deposition in diabetic kidneys (Srivastava et al., 2020a). Unlike ACEi, an ARB, which failed to protect the diabetic kidney against fibrosis, did not modulate miRNAs and DDP-4 expression (Nagai et al., 2014; Srivastava et al., 2020a).

## Angiotensin Converting Enzyme 2/Angiotensin-(1–7)/Mas Receptor Axis in Diabetic Nephropathy

Studies performed in the past few decades have revealed the great complexity of the RAS and demonstrated that, in addition to the classical ACE/Ang II/AT1R axis, the RAS comprises other important, biologically active enzymes, peptides and receptors (Simoes e Silva et al., 2013; Forrester et al., 2018; Povlsen et al., 2020). In 2000, the discovery of ACE2, a zinc metalloprotease homologous to ACE, revealed a new pathway for the Ang II peptide metabolism (Donoghue et al., 2000; Tipnis et al., 2000; Hamming et al., 2007). ACE2 hydrolyzes Ang II to the heptapeptide Ang-(1–7) and converts Ang I to the nonapeptide Ang-(1–9), which in turn can be converted to Ang-(1–7) by ACE, limiting Ang II production (Figure 1). Ang-(1–7) is produced mainly through the action of ACE2, which has a greater affinity for Ang II than Ang I; thus, Ang II is the major substrate for Ang-(1–7) synthesis. However, when levels of Ang II are not sufficiently elevated, Ang-(1–7) can also be formed directly from Ang I via neprilysin (NEP) (Rice et al., 2004). Ang-(1–7) is degraded to Ang-(1–5) by the action of ACE. Ang-(1–7) binds to a specific G protein-coupled receptor, the Mas receptor (Santos et al., 2003) triggering intracellular mechanisms and functional events that oppose many of the deleterious effects of Ang II, to the point that the ACE2/Ang-(1–7)/Mas receptor is considered the counterregulatory axis of ACE/Ang II/AT1R (Simoes E Silva and Teixeira, 2016; Rodrigues Prestes et al., 2017). In diabetes, an imbalance between the Ang II and Ang-(1–7) systems is indeed associated with vascular dysfunction, inflammation and fibrosis (Simoes e Silva et al., 2013; Srivastava P. et al., 2019).

### Angiotensin Converting Enzyme 2

ACE2 is an 805 amino-acid type 1 integral membrane glycoprotein (110–120 kDa) that consists of an extracellular domain, a transmembrane region and an intracellular tail. The extracellular domain of ACE2 contains a single active catalytic domain, unlike ACE, which consists of two catalytic domains (Donoghue et al., 2000; Tipnis et al., 2000; Batlle et al., 2012). ACE2 is mainly a tissue enzyme that is expressed at high levels in the kidneys, testes, intestine and heart (Donoghue et al., 2000; Tipnis et al., 2000) but can also be found in the lungs, liver, brain and pancreas. Unlike ACE, its levels in plasma are relatively low. A soluble form of ACE2 has been found in the circulation, in urine and in cerebrospinal fluid.

Studies have suggested that ACE2 has a renoprotective role in experimental renal diseases, including DN, particularly in combination with decreased ACE activity (Ye et al., 2004), because it enhances the degradation of Ang II (Batlle et al., 2012). Reduced expression of glomerular ACE2, coupled with increased expression of ACE, has been found in type 2 diabetic *db/db* mice, which favors excessive Ang II accumulation and its deleterious effects (Ye et al., 2006). After diabetic mice were treated with a specific ACE2 inhibitor, ACE increased further. In the same study, immunogold electron microscopy, used to identify the ultrastructural localization of ACE2 and ACE in the glomeruli of diabetic mice, showed that ACE2 was predominantly localized in podocyte foot processes, whereas ACE was expressed in glomerular endothelial cells. This finding suggested that the presence of ACE2 in podocytes could play an important counterregulatory role by preventing glomerular Ang II accumulation and the Ang II-mediated increase in glomerular permeability that results in the development of albuminuria (Ye et al., 2006). In this regard, the selective overexpression of human ACE2 in the podocytes attenuated the development of nephropathy in mice with streptozotocin-induced type 1 diabetes, and compared with wild-type diabetic mice, these mice experienced less glomerular injury, a delay in developing albuminuria, a blunted decrease in the podocyte markers nephrin and synaptopodin, and protection against podocyte loss (Nadarajah et al., 2012). On the other hand, pharmacological ACE2 inhibition worsened albuminuria and glomerular mesangial matrix expansion in streptozotocin-induced diabetic mice, in association with increased glomerular and vascular ACE expression (Soler et al., 2007; Wysocki et al., 2017). Studies using recombinant ACE2 in rodents have demonstrated the ability of ACE2 to rapidly metabolize ANG II *in vivo* and to promote Ang-(1–7) formation (Batlle et al., 2012). However, recombinant ACE2 induced an increase in plasma ACE2 activity but did not affect urinary ACE2, and failed to protect mice against the development of DN, indicating that when the augmentation of ACE2 activity is limited to the circulation, it is not sufficient to provide renoprotection because ACE2 needs to reach the urinary space to be effective (Wysocki et al., 2017). The fact that there was no increase in urinary ACE2 activity was attributed to the lack of glomerular filtration of recombinant ACE2, because of its large molecular size. Recently, shorter forms of ACE2, which are enzymatically active and can be filtered and delivered to the kidney, have been generated (Wysocki et al., 2019). They would enhance the formation of Ang-(1–7) from Ang II and could be a potential therapeutic approach for kidney diseases, including DN.

### Angiotensin-(1–7)

A large body of studies has shown that the biological actions of Ang-(1–7) through the Mas receptor are generally the opposite of those exerted by Ang II through its AT1R (Simoes E Silva and Teixeira, 2016; Rodrigues Prestes et al., 2017). Ang-(1–7) is formed mainly by Ang II via ACE2 and the balance between these two peptides within the RAS is greatly dependent on this enzyme (Marquez and Batlle, 2019). Beneficial effects of Ang-(1–7) have consistently been reported in experimental DN (Giani et al., 2012; Mori et al., 2014; Shi Y. et al., 2015; Zhang et al., 2015). The delivery of Ang-(1–7) by osmotic minipumps to Zucker diabetic fatty rats, a model of type 2 DN, thus caused a reduction in proteinuria, systolic blood pressure, and renal fibrosis, in association with decreased production of oxidative stress and inflammatory markers (Giani et al., 2012). Similarly, Ang-(1–7) treatment reduced oxidative stress, fibrosis and lipotoxicity in the kidneys of *db/db* mice (Mori et al., 2014). The infusion of Ang-(1–7) also attenuated the progression of streptozotocin-induced diabetic injury, limiting glomerulosclerosis, oxidative stress and cell proliferation (Zhang et al., 2015). Enhancing the Ang-(1–7) axis led to remarkable anti-inflammatory effects, resulting in the reduction of diabetes-induced leukocyte recruitment (Bossi et al., 2016). All these reported effects therefore made Ang-(1–7) a candidate therapeutic agent for DN.

### Cyclic Ang-(1–7) and Renoprotection in Diabetic Nephropathy

Ang-(1–7) has a short half-life in plasma, due to rapid *in vivo* catabolism by ACE and other proteases (Yamada et al., 1998), which is a limiting factor for its use for clinical purposes. Through the thioether cyclization method, a modified lanthipeptide cyclic (c)-Ang (1–7) was generated in which the amino acids Tyr4 and Pro7 were replaced with a D,L lanthionine (dAla-S-Ala) (Kluskens et al., 2009; Kuipers et al., 2019). The thioether-cyclized Ang-(1–7) provided enhanced resistance against proteolytic degradation in the circulation, with improved activity compared to the linear counterpart (Kluskens et al., 2009; de Vries et al., 2010). The higher resistance of cAng-(1–7) enables the use of lower doses and possibly less frequent administration than would be necessary with the linear peptide (Kluskens et al., 2009). Another advantage of the thioether-bridged cAng-(1–7) is that, unlike linear Ang-(1–7), it offers the possibility of oral and pulmonary delivery (de Vries et al., 2010). The lantipeptide cAng-(1–7) stimulates the Mas receptor, maintaining the receptor profile of the linear Ang-(1–7), specifically, as indicated by evidence that its vasodilating activity was abolished or decreased by the Mas receptor agonists D-Prot7 and D-Ala7 (Kluskens et al., 2009). In both mice with streptozotocin-induced diabetes and *db/db* mice, cAng-(1–7) caused an increase in insulin levels and reduced blood glucose levels, indicating the therapeutic potential that cAng-(1–7) has for treating type 1 and 2 diabetes (Kuipers et al., 2019).

Using BTBR *ob/ob* diabetic mice, a model that reproduces characteristic features of human type 2 DN better than other murine models, remarkable renoprotection was obtained after subcutaneous injections of cAng-(1–7) (Cassis et al., 2019a). Cyclic Ang (1–7) treatment, started when mice had already developed albuminuria, significantly limited the progressive increase in albuminuria that was observed in untreated BTBR *ob/ob* mice. Notably, we found that cAng (1–7) had as strong an antiproteinuric effect as the ACE inhibitor lisinopril, which was used for comparison, and limited glomerular fibrosis and inflammation even better than lisinopril (Cassis et al., 2019a). To uncover the mechanisms underlying the strong antiproteinuric effect of cAng-(1–7), we focused on podocytes and the glomerular endothelium because of their key role in maintaining an intact glomerular filtration barrier in DN (Weil et al., 2012; Siddiqi and Advani, 2013). cAng-(1–7) ameliorated the defective expression in podocytes of nephrin–the slit diaphragm protein that preserves slit pore integrity and renal filtration capacity–and nestin–a protein involved in the organization of the cytoskeleton–and limited podocyte loss, similar to the ACE inhibitor. cAng (1–7) was better at counteracting glomerular capillary rarefaction, a hallmark of advanced DN (Eleftheriadis et al., 2013), than lisinopril. The beneficial effects of cAng (1–7) on the glomerular endothelium were also revealed by electron microscopy analysis showing a reduction of vacuolization and improvement in the loss of endothelial fenestration. These data indicate that podocytes and glomerular endothelial cells are important targets of the renoprotective effects displayed by cAng-(1–7) in experimental diabetes. When cAng (1–7) was combined with lisinopril, the renoprotective action was additive, with a superior anti-proteinuric effect than ACE inhibitor had alone, along with better preservation of podocyte proteins and glomerular capillaries. Thus, cAng-(1–7), added to a background of chronic ACE inhibition, may provide a therapeutic opportunity for those diabetic patients who benefit less from ACE inhibitors.

## Effects of Sirtuins in Diabetic Nephropathy

Sirtuins are an evolutionarily conserved family of seven NAD^+^ -dependent deacetylases that reside in different subcellular compartments and regulate many physiological processes, including energy production, metabolism, mitochondrial biogenesis, stress resistance, inflammation and longevity (Haigis and Sinclair, 2010; Guarente, 2011; Morigi et al., 2018).

### Sirtuin-1

Of the seven sirtuins, sirtuin-1 has been one of the most extensively investigated in kidney diseases (Kong et al., 2015), and its renoprotective effects have been consistently demonstrated in experimental DN (Wang et al., 2019). It is localized in the nucleus and exerts its biological effects through the deacetylation of histones and transcription factors relevant for kidney disease progression, including p53, NF-kB, FOXO4, STAT3, PGC-1alpha, and consequently regulating their activities (Morigi et al., 2018; Wang et al., 2019). Sirtuin-1 protein expression was reduced in podocytes and in glomerular cells of human diabetic kidneys (Chuang et al., 2011).

#### Role of Sirtuin-1 on Podocytes

The podocyte-specific loss of sirtuin-1 reduced podocyte numbers, exacerbated albuminuria, and accelerated renal disease progression in diabetic mice (Chuang et al., 2014; Liu et al., 2014). There is evidence that sirtuin-1 is necessary for the preservation of cytoskeleton integrity and podocyte survival (Motonishi et al., 2015; Nakatani and Inagi, 2016). Analyses of isolated glomeruli from podocyte-specific sirtuin-1 knockout mice after the induction of a non-diabetic injury revealed severe morphological changes in podocytes, with foot process effacement and cytoskeleton derangement, in association with reduced expression of podocyte proteins, such as nephrin, nestin and Wilm’s tumor 1 protein (WT-1) (Motonishi et al., 2015). The mechanisms responsible for podocyte dysfunction after the loss of sirtuin-1 were found to be dependent on the inactivation of cortactin, an actin-binding protein that regulates the assembly, polymerization and stabilization of F-actin in different cell types, including podocytes. Indeed, sirtuin-1 deacetylated cortactin and enhanced cortactin activity, favoring localization in the cytoplasm and interaction with actin fibers, which are essential for maintaining the actin cytoskeleton (Motonishi et al., 2015). The induction of sirtuin-1 overexpression, specifically in podocytes, or treatment with the specific sirtuin-1 agonist BF175, in OVE26 type 1 diabetic mice, reduced albuminuria and attenuated diabetes-induced podocyte loss and oxidative stress, providing evidence that sirtuin-1 protects against diabetic disease (Hong et al., 2018). Sirtuin-1 renoprotection was mediated through PGC-1 alpha, the master regulator of mitochondrial function that, once deacetylated, protects podocytes against high glucose-induced oxidation and mitochondrial dysfunction (Hong et al., 2018). Some data suggest that in DN there is complex functional interplay between proximal tubules and glomeruli, regulated by sirtuin-1 (Hasegawa et al., 2013). The targeted deletion of sirtuin-1 in the proximal tubules of diabetic mice led to reduced levels of sirtuin-1 and high expression of the tight junction protein claudin-1 in podocytes, which led to the initiation of albuminuria and the development of renal function impairment (Hasegawa et al., 2013). To provide one potential explanation for these results, it was shown that proximal tubular cells exposed to high glucose concentrations *in vitro* secrete less nicotinamide mononucleotide (NMN), which lowers sirtuin-1 in podocytes and upregulates claudin-1 expression (Hasegawa et al., 2013). There is also some evidence that sirtuin-1 and the RAS interact, and this supports the hypothesis that sirtuin-1 is an important therapeutic target in DN. Sirtuin-1 activates the ACE2 promoter, thus favoring the production of Ang-(1–7) and its positive effects (Clarke et al., 2014; Mori et al., 2014). Ang-(1–7) increases sirtuin-1 expression, whereas Ang II has the opposite effect. In podocytes exposed to Ang II the expression of sirtuin-1 actually decreased, concomitant with the acetylation of p53, a pathway involved in podocyte apoptosis. Treating diabetic mice with an ARB that reduced albuminuria and protected podocytes against apoptosis and loss was associated with increased sirtuin-1 activity and reduced p53 acetylation in the kidneys (Gu et al., 2016).

#### Role of Sirtuin-1 on Endothelial Cells

It has been shown that sirtuin-1 regulates the angiogenic activity of endothelial cells and a specific deletion of its deacetylase activity in endothelial cells aggravated capillary rarefaction in a model of renal interstitial fibrosis (Potente et al., 2007; Kida et al., 2016). There is evidence that hyperglycemia-induced endothelial dysfunction was associated with sirtuin-1 downregulation and overexpression of vasoactive and profibrotic factors, such as endothelin-1 and TGF-β (Mortuza et al., 2015). Sirtuin-1 overexpression prevented glucose-induced increased endothelial permeability and extracellular matrix protein production *in vitro*. In addition, sirtuin-1 overexpressing transgenic mice with diabetes exhibited ameliorated albuminuria and kidney fibrosis (Mortuza et al., 2015). As for the mechanism(s) underlying the protective role of sirtuin-1 in diabetes-induced endothelial dysfunction, a recent study showed that sirtuin-1, through its deacetylase activity, suppresses the capacity of the 66-kDa Src homology two domain-containing protein (p66Shc) to induce vascular oxidative stress (Kumar et al., 2017). The p66Shc is a member of the Shc family of the adaptor proteins that acts as a redox enzyme for intracellular ROS generation. There is evidence that p66Shc is upregulated in cultured endothelial cells exposed to high glucose and in the vascular endothelium of diabetic mice, and that it is responsible for the upregulation of miR-34a, an upstream epigenetic regulator of sirtuin-1 (Li et al., 2016). Actually, systemic infusion of miR-34a inhibitor or genetic ablation of endothelial miR-34a prevented downregulation of endothelial sirtuin-1 caused by hyperglycemia (Li et al., 2016). All the above findings indicate that interplay between sirtuin-1, p66Shc and miR-34a regulates oxidative stress-driven dysfunction of vascular endothelium in diabetes.

### Sirtuin-3 and Diabetic Nephropathy

**FIGURE 2:**
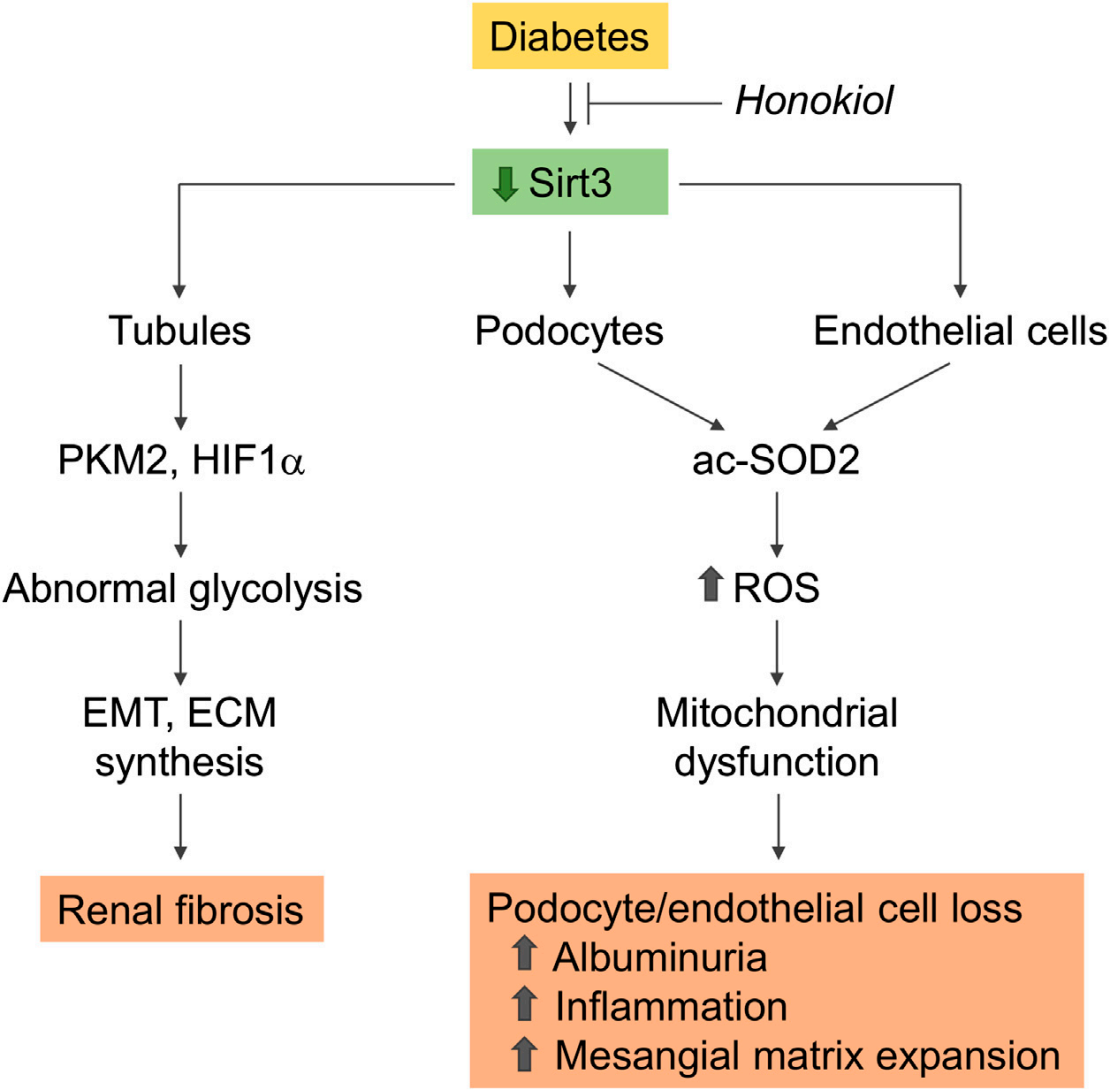
Role of sirtuin-3 dysregulation in kidney disease progression in diabetes. Sirtuin-3 is downregulated in the diabetic kidney. Reduced expression in podocytes and glomerular endothelial cells impairs SOD2 antioxidant activity as a consequence of enzyme acetylation, resulting in increased ROS generation, which promotes mitochondrial dysfunction and cell loss. These changes contribute to the development of albuminuria associated with inflammation and mesangial matrix expansion. Renal tubules with reduced sirtuin-3 undergo a metabolic reprogramming with a shift toward abnormal glycolysis, display EMT, and acquire a profibrotic phenotype. Rescuing sirtuin-3 by the specific activator honokiol prevents glomerular and tubule dysfunction and ameliorates diabetic nephropathy. acSOD2, acetylated SOD2; PKM2, pyruvate kinase isozyme M2; EMT, epithelial to mesenchymal transition; ECM, extracellular matrix.

Sirtuin-3, localized in the mitochondrial matrix, is the main mitochondrial NAD+ -dependent deacetylase that affects key mitochondrial processes, such as respiratory chain activity, the tricarboxylic acid cycle, ATP production, and antioxidant pathways (Ahn et al., 2008; Perico et al., 2016b). Changes in sirtuin-3 expression have a profound impact on the pathophysiology of several diseases, including metabolic syndrome, diabetes, and the aging processes (Benigni et al., 2016; Perico et al., 2016b; Benigni et al., 2019). In an *in vitro* model of diabetes, sirtuin-3 overexpression protected proximal tubular cells against high glucose-induced oxidative stress by enhancing the expression of antioxidant genes superoxide dismutase (SOD) and catalase (Jiao et al., 2016). Sirtuin-3 also protected endothelial cells from high glucose-induced cytotoxicity by modulating ROS production and oxidative stress through SOD deacetylation (Liu G. et al., 2015). There is evidence that sirtuin-3 mRNA expression is downregulated in kidney biopsies from DN patients (Wang X.X. et al., 2016). The kidneys of streptozotocin-induced diabetic CD-1 mice consistently exhibited a reduction in the sirtuin-3 protein, with the concomitant induction of a fibrogenic phenotype, which was exacerbated after sirtuin-3 suppression by the systemic administration of sirtuin-3 small interfering (si)RNA (Srivastava et al., 2018). The observation that in these mice the suppression of sirtuin-3 was associated with the induction of abnormal glycolysis, and that treatment with glycolysis inhibitors ameliorated renal fibrosis and restored sirtuin-3 levels as well, was taken to suggest that the restoration of sirtuin-3 could be a strategy for combating diabetes-associated kidney fibrosis through the inhibition of aberrant glycolysis (Srivastava et al., 2018) (Figure 2).

### Honokiol and Renoprotection in Diabetic Nephropathy

We recently demonstrated that renal sirtuin-3 mRNA expression was lower in type 2 BTBR *ob/ob* diabetic mice, in association with an impairment in its deacetylase activity toward SOD2, a major target of sirtuin-3, and was also associated with increased ROS production (Locatelli et al., 2020) (Figure 2). The selective activation of sirtuin-3 through the administration of honokiol, a natural biphenolic compound isolated from magnolia bark that has antioxidant, anti-inflammatory and anti-fibrotic properties, resulted in the attenuation of albuminuria and amelioration of glomerular injury (Locatelli et al., 2020). The anti-albuminuric effect of honokiol was associated with the amelioration of podocyte dysfunction and loss. In addition, honokiol limited glomerular capillary rarefaction, as revealed by immunofluorescence for CD-31, an endothelial cell marker, and the accumulation of Mac-2 positive monocytes/macrophages in the glomeruli. Sirtuin-3 activation with honokiol also translated into improvements in mitochondrial wellness in the glomeruli of diabetic mice through the activation of SOD2 and the restoration of the defective expression of PGC-1 alpha, a known regulator of mitochondrial homeostasis. The specific activation of sirtuin-3 is therefore effective in reducing diabetes-induced oxidative stress and providing protection for podocytes and more generally the glomerulus against diabetes-induced damage.

All the above evidence suggests that the pharmacological modulation of sirtuins is an attractive option for treating DN, and natural and synthetic sirtuin-activated compounds that have been tested in experimental kidney diseases (Morigi et al., 2018) are available for this purpose.

## Notch Signaling in Diabetic Nephropathy

The Notch signaling pathway comprises a family of four Notch transmembrane receptors (Notch1–4) and two different families of Notch ligands, namely Jagged (Jag1–2) and Delta-like (Dll1–4). The activation of this signaling requires cell–cell contact. Following ligand engagement, the Notch receptor is proteolytically cleaved by metalloproteases and the *γ*-secretase complex into the Notch intracellular domain (NICD), which enters the nucleus and associates with the DNA-binding protein CSL (CBF1/RBPjκ/Su(H)/Lag-1) and other transcriptional coactivators to trigger the transcription of target genes, such as hairy-enhancer of split (Hes) and Hes-related genes with the YRPW motif (Hey) (Kopan and Ilagan, 2009). Notch signaling is highly active during nephrogenesis, where it regulates nephron endowment and segmentation spatiotemporally through the differentiation of nephron progenitor cells into mature nephron cell types and patterning cell types within the collecting duct. Specifically, Notch2 signaling plays a key role early in nephrogenesis and is required in the acquisition of proximal nephron cell fates, including those of proximal tubules and podocytes (Mukherjee et al., 2019). Notch1 also contributes to nephrogenesis, albeit to a lesser extent than Notch2 (Surendran et al., 2010).

### Reactivation of Notch1 Signaling in Diabetic Nephropathy

In the normal kidney, Notch signaling is attenuated after birth and is inactive in the mature glomeruli of the adult kidney. *De novo* expression of active Notch1 in mature podocytes has been shown to induce apoptosis, which translates *in vivo* into the development of proteinuria and glomerulosclerosis (Niranjan et al., 2008). Active Notch1 leads to an increase in TGF-β1 transcription, which activates the Notch1 signaling pathway through the upregulation of Jag1. *De novo* expression of Notch pathway-related transcripts and the active Notch1 intracellular domain have been observed in the glomeruli and podocytes of murine and human diabetic kidneys (Niranjan et al., 2008). On the other hand, TGF-β1 is stimulated by diabetic states and plays an important role in the pathogenesis of DN (Ziyadeh, 2004). Thus, the interplay between Notch and TGF-β pathways in disease conditions is crucial in the regulation of podocyte apoptosis and can contribute to maintaining the damage.

#### Notch1 vs. Notch2

Proof that podocyte Notch signaling activation in DN plays a detrimental role comes from studies on the genetic knockdown of Notch signaling components. Diabetic mice with a podocyte-specific deletion of RBPj, which is essential for canonical Notch signaling, were partially protected against renal damage, exhibiting lower levels of albuminuria and less podocyte dedifferentiation and loss, accompanied by reduced TGF-β and vascular endothelial growth factor (VEGF) expression compared with wild-type mice with DN (Niranjan et al., 2008). In addition, the relative role of Notch1 vs. Notch 2 in podocytes during DN development was investigated in studies based on the specific genetic deletion or overexpression of each receptor (Sweetwyne et al., 2015). Podocyte-specific Notch1 deletion ameliorated DN, reducing albuminuria and mesangial expansion by preventing podocyte dedifferentiation and loss (Sweetwyne et al., 2015). In contrast, mice with podocyte-specific deletion of Notch2 were not protected against diabetic kidney disease development. Notch1-null podocytes exhibited preserved nephrin and podocin expression after TGF-β1 stimulation and were protected against growth factor-induced apoptosis. Moreover, glomeruli with podocyte-specific Notch1 deletion exhibited enhanced Notch2 expression, whereas Notch2 levels were lower in TGF-β1-stimulated podocytes with active Notch1, indicating that Notch1 regulates Notch2 in podocytes, both at baseline and after TGF-β1 treatment (Sweetwyne et al., 2015). Consistent with previous findings, podocyte-specific expression of the active Notch1 intracellular domain caused albuminuria and glomerulosclerosis, while mice with overexpression of the Notch2 intracellular domain did not exhibit phenotypic alterations (Sweetwyne et al., 2015). These studies highlighted the harmful role that Notch1 plays in inducing podocyte injury in diabetic kidney disease, while suggesting that Notch2 has a protective effect. Given the differential role of Notch1 and 2, it is likely that the loss of Notch1 and maintenance of Notch 2 in podocytes has a superior effect on glomerulosclerosis and proteinuria than the podocyte-specific loss of the pan-Notch regulator Rbpjk. In this context, the findings that higher glomerular Notch2 expression from diabetic mice that overexpressed podocyte-specific Mafb–a transcription factor that is essential for podocyte differentiation and foot process formation–ameliorated DN (Morito et al., 2014), and that pharmacological activation of Notch2 by an agonist mAb was beneficial against adriamycin-induced nephrosis (Tanaka et al., 2014) further support the hypothesis that this receptor has a protective effect on podocyte function and survival.

#### Triggers of Notch1 Signaling and Downstream Effectors

The Notch 1 signaling pathway has been recognized as playing a pathogenic role in DN through the induction of podocyte dysfunction and the loss of integrity of the glomerular filtration barrier, eventually resulting in proteinuria. The functional link between Notch1 activation and nephrin downregulation in podocytes, which is a hallmark of DN, is crucial to this event. Target transcripts that are induced by the active Notch1 intracellular domain include Snail, a transcriptional factor involved in EMT, which acts as a repressor of nephrin expression (Matsui et al., 2007). The Notch1/Snail pathway has been identified as the molecular mechanism underlying Ang II-induced nephrin downregulation in podocytes and the perpetuation of glomerular injury in experimental and human type 2 DN (Gagliardini et al., 2013). In Ang II-stimulated podocytes, the activation of Notch1 canonical signaling, through Hes1, upregulated the expression of Snail and its translocation into the nucleus, leading to nephrin downregulation. These effects were reversed by a *γ*-secretase inhibitor. In keeping with this, kidney specimens from either diabetic rats or humans exhibited a strong association between enhanced Snail protein signal and reduced nephrin protein expression. The Notch1/Snail pathway has clinical relevance, since its modulation by ACE inhibitors improved podocyte function and reduced overt proteinuria in diabetic patients (Gagliardini et al., 2013). *In vitro* studies have demonstrated that Ang II-induced Notch1 activation in podocytes was associated with the upregulation of TGF-β and VEGF, promoting apoptosis, and these effects were reversed by ARBs. Consistently, in the glomeruli of diabetic kidneys, the overexpression of TGF-β paralleled the increase in Jag1 and active Notch1 intracellular domain staining in podocytes. Ang II inhibition through telmisartan reduced albuminuria in Ins2 Akita diabetic mice by inhibiting TGF-β-associated activation of the Notch1 pathway (Koshizaka et al., 2012). In this context, another ARB, valsartan, also inhibited the activation of Notch, B-Cell CLL/Lymphoma 2 (Bcl-2) and p53 apoptotic pathways, and reduced apoptosis and podocyte detachment and loss in the glomeruli of mice with streptozotocin-induced diabetes (Gao et al., 2016). These findings suggest there is a link between Ang II and TGF-β in the activation of Notch1 signaling in podocyte loss in DN. Since TGF-β can also induce sustained Snail expression in podocytes via Notch1 (Sweetwyne et al., 2015), it is conceivable that the growth factor may also act as a mediator of Ang II-driven activation of the Notch1/Snail axis, leading to podocyte dedifferentiation. Another mechanism through which Notch triggers the onset of proteinuria is by promoting dynamin-dependent, raft-independent nephrin endocytosis (Waters et al., 2012).

Studies in cultured podocytes exposed to high glucose, mimicking diabetic conditions, enabled the identification of VEGF as a downstream effector of Notch1-induced podocyte dedifferentiation and apoptosis. VEGF, which is upregulated in the early stages of DN, is a direct trigger of nephrin repression and apoptosis in podocytes. The inhibition of Notch1 signaling by a *γ*-secretase inhibitor abrogated the high glucose-induced upregulation of VEGF, reduced nephrin expression and podocyte apoptosis and ameliorated proteinuria in diabetic rats (Lin et al., 2010). Another study has demonstrated there is interplay between the Notch1 and phosphatidylinositol 3-kinase (PI3K)/Akt pathways in regulating high glucose-induced podocyte apoptosis, suggesting that the balance between these two pathways may be important in the context of DN (Wang et al., 2014).

### Notch1 Signaling: A Therapeutic Target for Podocyte Protection in Diabetic Nephropathy

#### Pharmacological Modulation of Notch1 Signaling

**FIGURE 3:**
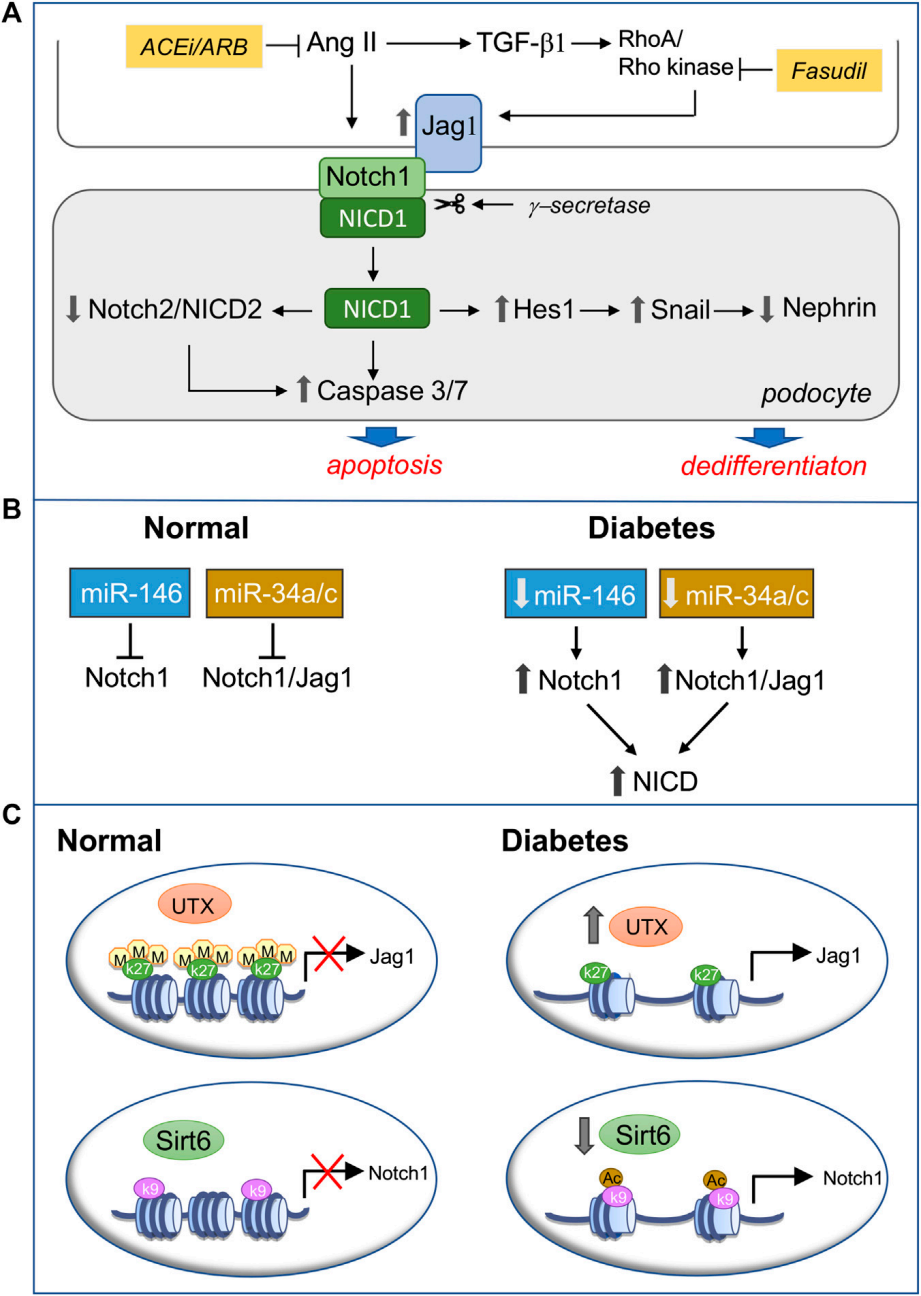
Modulation of podocyte Notch1 signaling in diabetic nephropathy (DN). **(A)** Pharmacological inhibition of active Notch1 inducers. Ang II and TGF-β drive podocyte injury in diabetes through the activation of Notch1 signaling. Following Jag1 engagement Notch1 is cleaved by *γ*-secretase and the released Notch1 intracellular domain (NICD1), via Hes1, induces sustained Snail expression, which represses nephrin, leading to podocyte dedifferentiation. On the other hand, NICD1 downregulates Notch2 and NICD2 and activates proapoptotic pathways, promoting podocyte apoptosis. Targeting Ang II by RAS inhibition and TGF-β-induced upregulation of Jag1 with the Rho kinase inhibitor fasudil prevents Notch1-mediated podocyte phenotypic changes and loss, ameliorating DN **(B**,**C)**. Epigenetic regulation of Notch1 signaling through miRNAs **(B)** or posttranslational histone modification **(C). (B)** In normal, mature, differentiated podocytes miR-146a and miRNA 34a/c prevent Notch1 signaling activation by targeting the 3′UTR of Notch1 or both Notch1 and Jag1, thus decreasing their mRNA and protein expression. Conversely, diabetes and hyperglycemia-induced downregulation of miR-146a and miRNA 34a/c result in Notch1 signaling activation in podocytes **(C)** In healthy podocytes the trimethylation of lysine residue 27 on histone protein H3 (H3K27me3) in the Jag1 promoter and the Sirt6-mediated deacetylation of lysine residue nine on histone protein H3 (H3K9) in the Notch1 promoter keep the Notch1 signaling pathway silent. In diabetes, reduced H3K27me3 – dependent on the overexpression of the demethylase UTX–and increased H3K9ac due to Sirt6 downregulation relieve the repression of Jag1 and Notch1, respectively, switching on Notch1 signaling in podocytes. ACEi, angiotensin converting enzyme inhibitor; ARB, angiotensin receptor blocker; UTX, Jumonji C domain-containing family: ubiquitously transcribed tetratricopeptide repeat on chromosome X.

The reactivation of Notch1 signaling in podocytes contributes to diabetic glomerulopathy, and its modulation can be achieved through the pharmacological inhibition of RAS (Koshizaka et al., 2012; Gagliardini et al., 2013; Gao et al., 2016), the standard therapy for CKD, including DN, and of Rho kinase, which mediates TGF-β-induced Jag1 expression in podocytes (Matoba et al., 2017) (Figure 3A). Type 2 diabetic mice treated with fasudil exhibited reduced albuminuria, urinary nephrin excretion and podocyte loss, which was associated with the downregulation of Jag1 and apoptotic markers in podocytes and less glomerular apoptosis (Matoba et al., 2017).

Preclinical studies have demonstrated that targeting Notch signaling via the genetic deletion of its components or the *γ*-secretase inhibitors ameliorated DN by having a protective effect on podocyte function and survival (Lin et al., 2010). However, *γ*-secretase inhibitors are nonspecific because they inhibit all *γ*-secretase complex-regulated intramembrane proteolyses of different substrates and fail to distinguish between individual Notch receptors. Moreover, concerns have been raised regarding adverse side effects on the gastrointestinal, immune and cutaneous systems, especially with long-term treatment (Wong et al., 2004; van Es et al., 2005). Notch receptor-specific inhibitors may overcome *γ*-secretase inhibitor-associated side effects, such as gastrointestinal tract toxicity, which depends on the dual inhibition of Notch1 and 2 receptors (Wu et al., 2010). Two different classes of Notch1 monoclonal antibodies are now available (Aste-Amezaga et al., 2010). Nanoparticle-based delivery of Notch1 monoclonal antibodies, which represses Notch signaling by locking the Notch1 receptors in a ligand-unresponsive state, is emerging as a promising, more targeted and efficient therapeutic strategy for treating cancer (Valcourt et al., 2020). The translatability of this tool for treating aberrant Notch1 signaling in DN remains to be established.

#### Islet-Like/Islet-Based Cell Therapy

Notch1 signaling in DN is emerging as a molecular target of the beneficial effects of cell therapy based on the administration of bone marrow-derived stem cells, which differentiate into islet-like cells in combination with microRNA 124a. Bone marrow-derived stem cells combined with miR-124a inhibited high glucose-induced podocyte apoptosis, concomitant with the repression of Notch1 activation, and ameliorated DN in type 2 diabetic rats (Sun et al., 2018). Islet transplantation consistently ameliorated albuminuria and podocyte ultrastructural changes in DN (He et al., 2018). However, these beneficial effects on renal injury and podocyte restoration were limited by the aberrant activation of Notch1 despite glycemic control, suggesting that the activation of this pathway by multiple factors can promote podocytopathy and disease progression and affect response to treatment. In this context, the overexpression of active Notch1 in podocytes is not limited to DN but is a common type of intracellular signaling underlying glomerulopathy in several proteinuric kidney diseases, where it strongly correlates with glomerulosclerosis (Murea et al., 2010).

#### Epigenetic Regulation of Notch1 Signaling

An alternative way of modulating Notch1 dysregulation in DN has been suggested by the recent discovery of the epigenetic regulation of Notch1 and/or its ligand Jag1 (Figures 3B,C).

#### MicroRNAs

Notch1 is a direct target of miR-146a, which is highly expressed in healthy podocytes, protecting them against diabetic injury. In contrast, miR-146a-deficient mice exhibited accelerated glomerulopathy and albuminuria following streptozotocin-induced hyperglycemia. Consistently, the downregulation of miR-146a in the glomeruli of both diabetic human and mouse kidneys correlated with glomerular damage and with a faster decline in renal function and paralleled the upregulation of Notch1 and ErbB4, a member of the epidermal growth factor receptor (Lee et al., 2017). Other miRNAs, including the miR-34 family members miR-34a and miR-34c, have been identified as upstream regulators of the Notch1 signaling pathway. Both miRNAs were downregulated in podocytes under hyperglycemic conditions, while their overexpression inhibited high glucose-induced podocyte apoptosis by directly targeting the 3′UTR of either Notch1 or Jag1, thus decreasing their mRNA and protein expression and blunting Notch1 signaling activation (Liu X. D. et al., 2015; Zhang et al., 2016).

#### Posttranslational Histone Modification

Chromatin dynamics control cell fate determination and the maintenance of a differentiated phenotype. Specifically, the trimethylation of lysine residue 27 on histone protein H3 (H3K27me3), is enriched at the promoter region of Jag1 and, by inhibiting Jag 1 transcription, restrains Notch pathway activity in adult differentiated podocytes (Majumder et al., 2018). The gain of the H3K27me3 mark is catalyzed by the histone methyltransferase enzyme, the enhancer of zeste homolog 2 (EZH2), while its loss depends on the activity of the Jumonji C domain-containing histone demethylases Jmjd3 and UTX. Notably, podocytes in glomeruli from humans with diabetic glomerulosclerosis exhibited reduced H3K27me3 concomitant with UTX overexpression, Jag1 upregulation, and nephrin loss. Moreover, the inhibition of Jmjd3 and UTX reduced albuminuria, podocyte foot process effacement, and Jag1 upregulation in diabetic mice, indicating that shifts in podocyte H3K27me3 levels may influence the development and outcomes of glomerular injury in DN (Majumder et al., 2018).

Chromatin remodeling and gene transcription are also regulated by histone acetylation/deacetylation, with deacetylated histones being associated with transcriptional repression. Sirtuin-6 is a member of the sirtuin family of class III NAD^+^-dependent histone deacetylases, which inhibits Notch signaling by deacetylating lysine residue nine on histone protein H3 (H3K9). Sirtuin-6 expression was reduced in the kidneys of type 1 and 2 diabetic mice, mainly in the podocytes, and in renal biopsies from DN patients. It correlated positively with estimated glomerular filtration rate and negatively with proteinuria and was associated with increased H3K9ac levels. Lower Sirt6 expression in high glucose-treated podocytes consistently paralleled the increased levels of H3K9ac in the promoters of Notch1 and Notch4 and the overexpression of Notch downstream target genes Hes1 and Snail. Furthermore, the activation of Notch signaling is part of the mechanism through which podocyte-specific loss of sirtuin-6 exacerbates podocyte injury and proteinuria in DN (Liu et al., 2017).

Altogether these findings suggest that the epigenetic regulation of Notch1 signaling through the modulation of either miRNA or posttranslational histone modification could be a novel strategy for preventing the reactivation of this developmental pathway in podocytes during glomerular disease and a potential therapeutic intervention that confers protection against DN.

## Thyroid Hormone Signaling

### Thyroid Hormone Signaling: A Critical Player in Diabetes-Induced Fetal Reprogramming

Thyroid hormone signaling plays a critical role in physiological growth and organ development. It is mediated by two main classes of thyroid hormone receptors (TRs) that regulate gene transcription: TR alpha (TRα) and TR beta (TRβ). In mammals, the predominant TR isoforms include TRα1, TRβ1, TRβ2, TRβ3, TRβ4. Other TR variants lack T3-binding capacity, and these are TRα2, TRα3 and TRαΔE6. TRβ is the predominantly adult isoform and regulates TH levels and the liver and kidney metabolism, and is also critical for the normal development of auditory and visual systems (Brent, 2012; Mourouzis et al., 2020). TRα1 is highly expressed in developing organs, including the heart, brain and kidney, and plays a key role in cell proliferation prenatally, while after birth it regulates differentiation in various cell types (Horn and Heuer, 2010; Pantos and Mourouzis, 2014; Benedetti et al., 2019). In the fetus, when the levels of the active form of TH L-triiodothyronine (T3) are low, TRα1 mainly acts as an apo-receptor (unliganded state) to repress adult genes (thus protecting the embryo from premature differentiation) and enhances cell proliferation and organ growth. In contrast, after birth when T3 levels increase, TRα1 switches to the holo-receptor (liganded state) to induce the expression of adult genes, thus promoting cell differentiation, physiological organ maturation and function.

Compared to the healthy population, diabetic patients exhibit lower T3 plasma levels (Wu et al., 2015) and a higher prevalence of thyroid dysfunction, suggesting the recurrence of the fetal profile, with low T3 levels. Several clinical studies have also shown that thyroid dysfunction and low T_3_ levels are strongly associated with worse renal clinical outcomes and increased mortality in diabetic patients (Zoccali et al., 2006; Lazzeri et al., 2012; Rhee, 2016). However, the etiogenesis underlying these phenomena remains poorly understood.

Recent studies in our lab have shown that podocytes and parietal epithelial cells in the glomeruli of patients and rats with DN re-expressed the fetal isoform TRα1, and that these cells were also positive for several fetal, mesenchymal and damage-related podocyte markers (Benedetti et al., 2019). Notably, the simultaneous re-expression of TRα1 and fetal markers in the glomerulus was observed in almost all of the common rodent models of DN (i.e. streptozotocin-induced type I diabetes, and the models of type II diabetes with a deficiency for leptin (*ob/ob* mice) or for leptin receptor (Zucker diabetic fatty rats). In rats with DN, we also observed that the glomerular expression of the TH-inactivating enzyme deiodinase 3 (DIO3) increased, and blood T3 levels decreased progressively, correlating inversely with the metabolic and renal disease worsening. In addition, human podocytes exposed to typical components of the diabetic milieu *in vitro* (high glucose and H_2_O_2_), exhibited markedly upregulated TRα1 and DIO3 expression. The adoption of this fetal profile of TH signaling was associated with cytoskeleton rearrangements, adult podocyte marker downregulation and fetal kidney marker upregulation, along with the induction of a maladaptive cell cycle, and TRα1-ERK1/2-mediated hypertrophy (Benedetti et al., 2019).

It is noteworthy that similar alterations in the TH-TRα1 axis are also observed in cardiomyocytes, another terminally differentiated and highly specialized cell type. In response to a wide range of stressful stimuli, cardiomyocytes adopt a fetal TH signaling profile and (at least partially) re-activate the fetal gene program, which eventually leads to structural alterations and the deterioration of organ function. It has been shown that during adrenergic injury the unliganded TRα1 induces the adoption of a fetal pattern of myosin isoform expression and radical phenotypical changes in the structure, shape and size of neonatal cardiomyocytes (Pantos et al., 2007). Similarly, inhibiting T3 binding to the TRα1 receptor delayed cardiac myoblast differentiation, while enabling the T3-TRα1 binding reversed all the aforementioned phenotypical changes (Pantos et al., 2008). Our ongoing studies have shown that cardiac TH signaling was also altered in diabetic rats, and these alterations were associated with molecular and phenotypical changes in the left ventricle.

**FIGURE 4:**
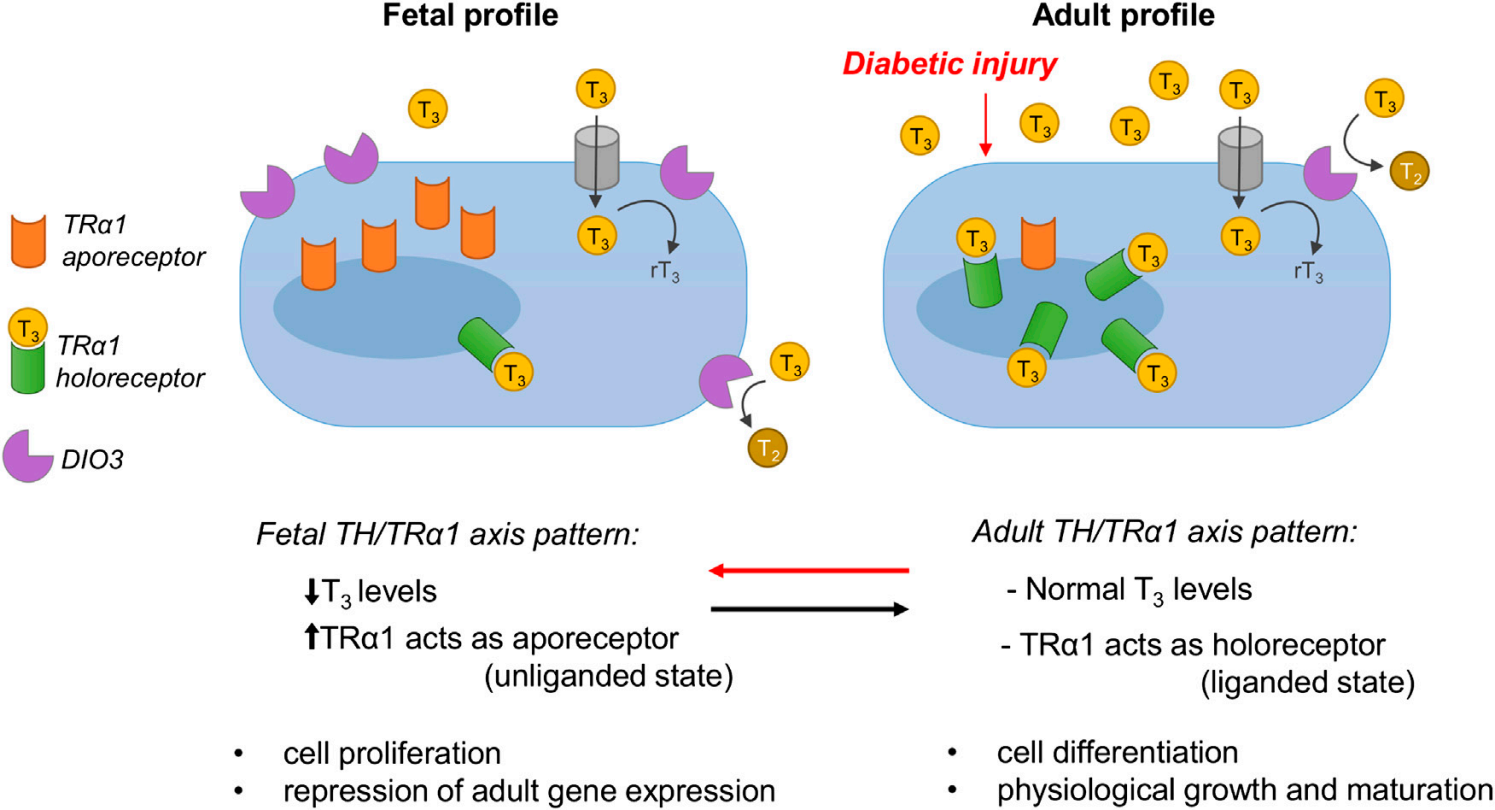
In the fetus, when the levels of the active form of TH L-triiodothyronine (T3) are low, TRα1 mainly acts as an apo-receptor (unliganded state) to repress adult genes (thus protecting the embryo from premature differentiation) and enhances cell proliferation and organ growth. In contrast, after birth when T3 levels increase, TRα1 switches to the holo-receptor (liganded state) to induce the expression of adult genes, thus promoting cell differentiation, physiological organ maturation and function. In adult life, local T_3_ availability is controlled by the T_3_-inactivating enzyme DIO3, which converts excessive T_3_ into rT_3_ and T_2_. In response to diabetic injury, systemic T_3_ levels drop markedly, and TRα1 and DIO3 are overexpressed locally, resulting in the coordinated adoption of the fetal ligand/receptor relationship profile (i.e., low T_3_ availability/high local TRα1). Apo-TRα1 binds DNA and represses the transcription of target adult genes (as happens in the fetus), leading to cell dedifferentiation, metabolic and structural remodeling, and cell cycle reactivation. The figure is modified from Benedetti et al., 2019.

In light of the above data, which demonstrate the existence of a causal link between the reduction in TH availability and the reactivation of developmental pathways in adulthood, and considering the crucial regulatory role of TH signaling in development and metabolism, we hypothesize that the TH/TRα1 axis is a key regulator of the reactivation of the cell developmental program (defined as fetal reprogramming, FR) in terminally differentiated and highly specialized cells (Figure 4).

### Adopting a Fetal Thyroid Hormone Signaling Profile: Adaptation, Maladaptation or Therapeutic Opportunity?

So far, our data suggest that the fetal profile of TH signaling (characterized by low T3 systemic levels, TRα1 in the apo-receptor state and increased DIO3 activity) in the diabetic kidney triggers podocytes to dedifferentiate and re-activate fetal genes, re-enter the cell cycle and increase DNA content and cell size. Although the role of TH signaling in the pathobiology of the stressed kidney is clear, the biological significance of this response, especially in humans, remains unknown.

One hypothesis we can put forward is that the reactivation of fetal TH signaling is an adaptive response of the tissue to diabetes-induced chronic stress to enable non-proliferating cells to reach a lower energy state and/or to allow for compensatory growth. Alternatively, it could be a maladaptive response of the tissue that leads to phenotypical changes that are more detrimental than beneficial. The insufficient availability of data in DN limits the formulation of robust hypotheses about the biological significance of the adoption of fetal TH signaling in the diabetic kidney. Nevertheless, analyzing data and paradigms from other organs may help us to better understand the causal rationale behind this response. Generally, low T3 levels initially provide a metabolic benefit to stressed organs. In the diabetic heart, for example, the local reduction of T3 levels triggers a metabolic switch that is associated with remodeling of the contractile machinery, which includes a switch from the expression of proteins that consume high levels of energy to energy-saving ones (Rajagopalan and Gerdes, 2015). In addition to the metabolic/energetic benefit, low T3 levels also enable the proliferation of damaged cells in some tissues that are endowed with high regenerative capacities. This is clearly observed in skeletal muscles, which in mice can regenerate through satellite cell amplification after acute injury (Dentice et al., 2010; Dentice et al., 2014). The regeneration process begins with a drastic DIO3-mediated reduction of local T3 concentrations (Dentice et al., 2014), which allows for cell proliferation and is immediately followed by a concomitant downregulation of DIO3 and upregulation of DIO2 expression, thus leading to a renewed increase in T3 levels, enabling cell differentiation (Dentice et al., 2010). This strategy is effective for organs that can regenerate through cell proliferation and differentiation (at least in response to acute injuries). However, for terminally differentiated and highly specialized cells, such as podocytes and cardiomyocytes, which cannot proliferate without affecting organ integrity and function, another strategy was selected. In order to cope with the increased workload that results from cell loss, these cells dedifferentiate and increase their genome content (polyploidization) and cell size (hypertrophy). For these coordinated phenotypical alterations to occur, the fetal profile of TH signaling (characterized by low T3 systemic levels, TRα1 in the apo-receptor state and/or increased DIO3 activity) must be recapitulated. Even though this is beneficial for organ function (at least in the early stages of the disease), the persistent lack of T3 induces extensive cell dedifferentiation and maladaptive proliferation, the reactivation of several developmental pathways, and pathological growth and structural remodeling in damaged tissue. Several studies in experimental hypothyroidism support this concept. In diabetes, hypothyroidism induces the dedifferentiation and/or transdifferentiation of pancreatic *β*-cells, and these phenomena (instead of apoptosis) have been proposed as putative explanations for pancreatic *β*-cell loss (Moin and Butler, 2019). Notably, the simultaneous overexpression of TRα1 and the administration of T3 enhanced cell cycle progression and proliferation, leading to the reprogramming of pancreatic cells into insulin-producing cells, in both the rat *β*-cell line and in an animal model of STZ-induced diabetes (Furuya et al., 2010). These findings are consistent with our studies on the diabetic kidney (Benedetti et al., 2019) and indicate that the adoption of a fetal TH signaling profile is associated with cell dedifferentiation and loss and pathological growth.

Regardless of whether these phenomena should be considered adaptations or maladaptations, they can be exploited as therapeutic opportunities: controlling these pathways spatiotemporally could in fact be a strategy for directing the regeneration of damaged tissues. Administering T3 to pharmacologically modulate the TH-TRα axis has indeed exhibited exceptional therapeutic potential in various diabetic organs (Furuya et al., 2010; Lin and Sun, 2011; Mourouzis et al., 2013) and in *in vitro* models (Furuya et al., 2010; Benedetti et al., 2019). In diabetic milieu-injured podocytes, T3 treatment completely reversed the fetal phenotype and subsequent pathological alterations by upturning changes in TH signaling, promoting re-differentiation, and restoring normal cellular morphology (Benedetti et al., 2019). In the kidneys of patients with chronic kidney disease, TH treatment improves renal function (Shin et al., 2012, Shin et al., 2013), while in our ongoing models of diabetes, T3 reverses FR by promoting re-differentiation and reducing hypertrophy, and improves renal structure.

Nevertheless, translating this strategy into clinical practice will not be straightforward: the high doses of T3 that need to be administered to achieve therapeutic effects under conditions of systemic hypothyroidism lead to various adverse effects (Collet et al., 2012; Ali Rajab et al., 2017; Pantos and Mourouzis, 2018). Thus, to maximize therapeutic efficacy while minimizing possible adverse effects, future therapeutic strategies should use drug delivery systems that can target and deliver the drug to injured cells only. Alternatively, new thyromimetics with a higher affinity for TRα1 and less susceptibility to inactivation need to be produced to allow for more efficient receptor activation and to drastically reduce the high dose-related adverse effects. We are currently working in both directions to ensure the success of the most clinically promising option.

## Sodium-Glucose Cotransporter 2

SGLT2, which is located on the apical membrane of renal tubular epithelial cells, is the principal contributor to the reabsorption of filtered glucose, and SGLT2 inhibitors are now a well-defined class of anti-hyperglycemic agents for type 2 diabetes. These drugs block renal reabsorption of glucose, promoting glycosuria and lowering blood glucose (Vallon and Thomson, 2017). In addition, SGLT2 inhibitors have direct effects on glomerular hemodynamics, which are important for renoprotection in DN. In diabetes, because of a high filtered load of glucose, reabsorption of glucose and sodium is increased in the proximal tubule via SGLT2, with a resulting diminished delivery of sodium to the macula densa. These effects reduce the tubuloglomerular feedback signal, causing constriction of the adjacent efferent arteriole, and dilatation of the afferent arteriole, leading to increases in intraglomerular pressure and single nephron GFR. The inhibition of SGLT2 increases the delivery of sodium to the macula densa, restoring tubuloglomerular feedback and promoting afferent arteriolar constriction, which results in reduced intraglomerular pressure and hyperfiltration (Skrtic and Cherney, 2015; DeFronzo et al., 2017; Perico et al., 2017; Toto, 2017) would translate into lowered albuminuria and reduced progression of the diabetic kidney disease. Several kidney and cardiovascular outcome studies in type 2 diabetes have indeed demonstrated that there are important advantages to using SGLT2 inhibitor therapy, including mortality benefits (for review see (Alicic et al., 2019) (Kanduri et al., 2020). In experimental diabetes SGLT2 inhibitors controlled hyperglycemia and limited albuminuria and renal damage, including glomerular mesangial matrix accumulation and interstitial fibrosis, through combined effects on glomerular hemodynamics and the inhibition of inflammation and oxidative stress (Gembardt et al., 2014; Terami et al., 2014; Vallon et al., 2014; Wang et al., 2017). SGLT2 inhibition also prevented podocyte injury and loss (Wang et al., 2017). There is evidence that in addition to tubular epithelial cells, SGLT2 is expressed in glomerular cells, and that SGLT2 inhibitors may exert tubular SGLT2-independent reno-protective effects (Cassis et al., 2018; Maki et al., 2019). The expression of SGLT2 protein has been demonstrated in cultured mesangial cells, and was upregulated by exposure to high glucose (Maki et al., 2019). Moreover, in *db/db* mice with type 2 diabetes, a low dose of SGLT2 inhibitor–which, unlike a higher dose, did not affect hyperglycemia and glycosuria–was still able to reduce albuminuria and mesangial expansion in the same way as a higher dose (Maki et al., 2019). Through *in vitro* and *in vivo* experiments, we have shown that SGLT2 is also expressed in mouse podocytes and that its level was increased by albumin overload, depending on NF-kB activation (Cassis et al., 2018). Further, we showed that SGLT2 inhibitor limited proteinuria and protected mice with protein-overload proteinuria against podocyte dysfunction and loss, and that SGLT2 inhibitor directly targeted podocytes through the maintenance of actin cytoskeleton architecture (Cassis et al., 2018). All the above evidence indicates that SGLT2 inhibitors, through their pleiotropic effects, independently of their glucose-lowering property, may provide renoprotection not only in diabetic but also non-diabetic CKD.

## Hypoxia-Inducible Factor-1

Chronic hypoxia has been recognized as an important signaling pathway driving diabetic kidney disease (Hesp et al., 2020). Emerging evidence indicates that many of the renoprotective benefits of SGLT2 inhibitors may be due to their action on hypoxia-inducible factor (HIF)-1 (Bessho et al., 2019; Packer, 2020), a heterodimeric transcription factor that plays a key role in cellular adaptation to different oxygen concentrations (Patten et al., 2010; Packer, 2020). It is composed of an oxygen-sensitive *α*-subunit (HIF-1α) and a constitutively expressed *ß*-subunit (HIF-1β). In normoxic conditions, HIF-1α subunit is continuously produced in the cytosol but rapidly degraded; it is hydroxylated at specific proline residues by prolyl-4-hydroxylase domain (PHD) proteins, allowing for recognition by von Hippel-Lindau-(VHL)-E3 ubiquitin ligase complex, which targets HIF-1α for proteasomal degradation. During hypoxia, the degradation process is suppressed and HIF-1α is transferred into the nucleus to form, with the *ß*-subunit, an active heterodimer that binds to hypoxia response elements (HRE) in the promoter regions of target genes involved in different processes, including erythropoiesis, glycolysis, angiogenesis, oxidative stress and fibrogenesis (Haase, 2006; Packer, 2020). Notably, in addition to hypoxia, nonhypoxic factors such as high glucose, Ang II, TGF-β and ROS–all of which mediate renal damage in diabetes–promote HIF-1 activation (Macconi et al., 2014; Nayak et al., 2016). HIF-1 is *per se* implicated in the regulation of the above mediators, so it has been proposed that there is a feedback loop through which HIF-1 mediates the initiation and progression of diabetes-induced renal damage (Nayak et al., 2016). It has been reported that the activation of HIF-1 signaling by hypoxia promoted fibrosis. Thus HIF-1α enhanced EMT transition in renal epithelial cells *in vitro,* and genetic ablation of epithelial *Hif-1α* reduced tubulointerstitial fibrosis in a mouse model of kidney fibrosis (Higgins et al., 2007). Increased expression of HIF-1 and its target genes has been found in fibrotic areas of microdissected kidney tissues from DN patients (Higgins et al., 2007), and the upregulation of HIF-1α has been detected in hypertensive DN kidneys of mice with renal fibrosis (Jiao et al., 2018). Moreover, HIF-1α blockade through treatment with a HIF-1 inhibitor ameliorated glomerular hypertrophy, mesangial matrix expansion and fibrosis in diabetic OVE26 mice (Nayak et al., 2016). Based on *in vitro* and *in vivo* experiments, recent studies have proposed HIF-1 as a therapeutic target for an SGLT2 inhibitor for DN (Bessho et al., 2019; Packer, 2020). In cultured tubular epithelial cells, an SGLT2 inhibitor reduced hypoxia-induced HIF-1α protein expression and its target genes by reducing mitochondrial oxygen consumption (Bessho et al., 2019). In diabetic *db/db* mice, treatment with the SGLT2 inhibitor attenuated cortical tubular HIF-1α expression, tubular injury and interstitial fibrosis (Bessho et al., 2019). There is also evidence that in type 2 diabetes, SGLT2 inhibitors enhanced nutrient deprivation signaling through the upregulation of AMPK and SIRT1, which in turn act to suppress HIF-1α (Packer, 2020).

## Dipeptidyl Peptidase-4

Dipeptidyl peptidase-4, also known as CD26, is a ubiquitously expressed serine protease that cleaves several substrates, including the incretin hormones, glucagon-like peptide 1 (GLP–1) and glucose-dependent insulinotropic polypeptide (GIP), which regulate post-prandial insulin secretion (Röhrborn, 2015). DPP-4 inhibitors have been approved as antihyperglycemic medication for type 2 diabetes. DPP-4 inhibitors are oral, weight neutral, well tolerated blood glucose-lowering drugs with a low risk of hypoglycemia and proven cardiovascular safety (Gallwitz, 2019). Clinical studies have reported that some DPP-4 inhibitors used as monotherapy or added to ACE inhibitors/ARBs reduced albuminuria in diabetic patients without affecting other renal outcomes (see reviews (Penno et al., 2016; Coppolino et al., 2018; Taylor and Lam, 2020). However, there are no definitive data that would make it possible to establish whether DPP-4 inhibitors confer renoprotection on type 2 patients (Hanssen and Jandeleit-Dahm, 2019).

The role of DPP-4 and the effects of DPP-4 inhibitors in diabetic kidney disease have been reviewed recently, with a focus on linagliptin (see (Kanasaki, 2018; Gupta and Sen, 2019). In the healthy rat kidney, DPP-4 is expressed in proximal tubular cells and in the glomerulus, mainly in podocytes. In humans, glomerular expression of DDP-4 was only detected under pathological conditions. Consistent with this, *in vitro* studies have reported DPP-4 induction in human podocytes and glomerular endothelial cells in response to inflammatory cytokines and high glucose (Kanasaki, 2018). In experimental DN the DPP-4 inhibitor linagliptin reduced albuminuria and ameliorated glomerulosclerosis and interstitial fibrosis, independently of glucose control (Kanasaki, 2018). The renoprotective effects were associated with the attenuation of podocyte dysfunction and loss (Sharkovska et al., 2014; Takashima et al., 2016) and the inhibition of EndMT (Kanasaki et al., 2014). The molecular mechanisms underlying DPP4-induced EndMT have been elucidated by *in vitro* studies that showed that DPP-4 interacts with the integrin β1, causing TGF-βR heterodimer formation and the consequent activation of TGF-β signaling. The DPP-4/integrin β1 complex can also downregulate VEGFR2 while upregulating VEGFR1, thus favoring EndMT (Shi S. et al., 2015). In diabetic kidneys DPP-4 is overexpressed in endothelial cells with a mesenchymal phenotype, concomitant with the downregulation of miR-29s. By restoring miR-29s, which target DPP-4, linagliptin inhibited DPP-4 overexpression and its interaction with integrin β1, thus reducing TGF-β-induced EndMT (Kanasaki et al., 2014; Shi S. et al., 2015). This effect is unique to linagliptin and not shared by other members of the gliptin family (Shi et al., 2016). Similarly to what has been observed in endothelial cells, linagliptin is able to reduce TGF-β signaling in proximal tubular cells under hyperglycemic conditions by inhibiting the interaction of DPP-4 with the cation-independent mannose 6-phosphate receptor (Gangadharan Komala et al., 2015). Altogether these findings suggest that the DPP-4 inhibitor linagliptin has a pleiotropic effect that is incretin- and glucose-lowering- independent, and which confers protection against kidney fibrosis in experimental DN through miRNA modulation and the inhibition of DPP-4 interaction with other proteins.

## Conclusion

Diabetes is a global health concern of epidemic proportions. About one-third of affected people develop diabetic nephropathy, a leading cause of end-stage kidney disease worldwide. There is an imperative need to identify novel therapeutic interventions with renoprotective effects for those diabetic patients who do not respond completely to standard therapy. In this review we first described four major signaling pathways that have emerged as mediators of podocyte/endothelial cell injury that contribute crucially to the pathogenesis of DN and can be targets for therapeutic interventions (Table 1). The development of cAng-(1–7), a modified peptide that is more peptidase resistant than the linear peptide, is particularly attractive for long-term treatment and has potential suitability for clinical use. The availability of natural compounds that increase sirtuin expression/activity in the diabetic kidney makes pharmacological modulation of sirtuins a novel strategy for treating DN. Notch1 and TH signaling pathways, which are abnormally activated in podocytes in DN, are targets for podocyte-directed therapy. Future drug delivery systems that can target and deliver the TH to injured cells, or new thyromimetics with a higher affinity for TRα1, may allow us to maximize the regenerative potential of TH signaling and minimize the high dose-related adverse effects. In addition, there are a number of different experimental therapies that could directly or indirectly target other discussed signalings. Actually, drugs that target podocytes or vasculature, such as SGLT2 inhibitors and DPP-4 inhibitors, as well as drugs that can modulate HIF activity, may lead to next-generation therapeutics that can efficiently mitigate diabetes complications in the kidney. Finally silencing of miRNAs that are found to directly contribute to the pathogenesis of DN, e.g., miR-21 (Kölling et al., 2017), miR-214 (Wang X. et al., 2016) or miR-184 (Zanchi et al., 2017), to name a few, or to induce changes in TH signaling (e.g., induction of the DIO3 (Di Girolamo et al., 2016), may provide a solid basis for the development of therapeutic solutions that can arrest or even reverse the structural and functional alterations of the diabetic kidney.

**TABLE 1:** Selected targetable signaling pathways in experimental diabetic nephropathy mentioned in this review.

Pathway	Intervention	Model	References
ACE2/Ang-(1‒7)/MasR	Podocyte-specific hACE2 overexpression	STZ-induced diabetes in mice	Nadarajah et al. (2012)
	Ang-(1‒7)	Zucker diabetic fatty rats, *db*/*db* mice, STZ-induced diabetes in mice	Giani et al. (2012), Mori et al. (2014), Zhang et al. (2015), Bossi et al. (2016)
	Cyclic Ang-(1‒7)	BTBR *ob*/*ob* mice, type 1 and type 2 diabetes mouse models	Cassis et al. (2019a), Kuipers et al. (2019)
Sirtuin-1	Podocyte-specific sirtuin-1 overexpression Sirtuin-1 agonist BF175	OVE 26 type 1 diabetic mice	Hong et al. (2018)
Sirtuin-3	Honokiol	BTBR *ob*/*ob* mice	Locatelli et al. (2020)
Notch	Podocyte-specific RBPj deletion	STZ-induced diabetes in mice	Niranjan et al. (2008)
	Podocyte-specific Notch 1 deletion	STZ-induced diabetes in mice	Sweetwyne et a. (2015)
	Podocyte-specific Mafb overexpression	STZ-induced diabetes in mice	Morito et al. (2014)
	γ-secretase inhibitor DAPT	STZ-induced diabetes in mice	Lin et al. (2010)
	histone demethylase inhibitor GSK-J4	*db*/*db* mice	Majumder et al. (2018)
Thyroid hormone	L-triiodothyronine (T3)	*db*/*db* mice	Lin and Sun (2011)
		high glucose-loaded podocytes	Benedetti et al. (2019)

## Author Contributions

All authors listed have made a substantial, direct, and intellectual contribution to the work and approved it for publication.

## Funding

CX’s research is funded by Euronanomed (an ERA-NET grant; 736/8221) and the Associazione per la Ricerca sul Diabete Italia.

## Conflict of Interest

The authors declare that the research was conducted in the absence of any commercial or financial relationships that could be construed as a potential conflict of interest.
